# Evaluation of electrical impedance tomography for determination of urinary bladder volume: comparison with standard ultrasound methods in healthy volunteers

**DOI:** 10.1186/s12938-018-0526-0

**Published:** 2018-07-13

**Authors:** Dorothea Leonhäuser, Carlos Castelar, Thomas Schlebusch, Martin Rohm, Rüdiger Rupp, Steffen Leonhardt, Marian Walter, Joachim O. Grosse

**Affiliations:** 1 Department of Urology, RWTH Aachen University Hospital, Pauwelsstraße 30, 52074 Aachen, Germany; 2 Philips Chair for Medical Information Technology (MedIT), RWTH Aachen University, Aachen, Germany; 3 Spinal Cord Injury Center, Heidelberg University Hospital, Heidelberg, Germany

**Keywords:** Electrical impedance tomography, Non-invasive, Urinary bladder volume, Ultrasound, Cystovolumetry

## Abstract

**Background:**

Continuous non-invasive urinary bladder volume measurement (cystovolumetry) would allow better management of urinary tract disease. Electrical impedance tomography (EIT) represents a promising method to overcome the limitations of non-continuous ultrasound measurements. The aim of this study was to compare the measurement accuracy of EIT to standard ultrasound in healthy volunteers.

**Methods:**

For EIT of the bladder a commercial device (Goe MF II) was used with 4 different configurations of 16 standard ECG electrodes attached to the lower abdomen of healthy participants. To estimate maximum bladder capacity (BCmax) and residual urine (RU) two ultrasound methods (US-Ellipsoid and US-L × W × H) and a bedside bladder scanner (BS), were performed at the point of urgency and after voiding. For volume reference, BCmax and RU were validated by urine collection in a weight measuring pitcher. The global impedance method was used offline to estimate BCmax and RU from EIT.

**Results:**

The mean error of US-Ellipsoid (37 ± 17%) and US-L × W × H (36 ± 15%) and EIT (32 ± 18%) showed no significant differences in the estimation of BCmax (mean 743 ± 200 ml) normalized to pitcher volumetry. BS showed significantly worse accuracy (55 ± 9%). Volumetry of RU (mean 152.1 ± 64 ml) revealed comparable higher errors for both EIT (72 ± 58%) and BS (63 ± 24%) compared to US-Ellipsoid (54 ± 25%). In case of RU, EIT accuracy is dependent on electrode configuration, as the Stripes (41 ± 25%) and Matrix (38 ± 27%) configurations revealed significantly superior accuracy to the 1 × 16 (116 ± 62%) configuration.

**Conclusions:**

EIT-cystovolumetry compares well with ultrasound techniques. For estimation of RU, the selection of the EIT electrode configuration is important. Also, the development of an algorithm should consider the impact of movement artefacts. Finally, the accuracy of non-invasive ultrasound accepted as gold standard of cystovolumetry should be reconsidered.

## Background

It is estimated that around one million people in Germany suffer from bladder dysfunction [1]. Dysfunctions of the storage and voiding function of the bladder are often associated with an overactive bladder with signs of urgency, frequent voiding and nocturia with or without urinary incontinence. Furthermore, neurological diseases and spinal cord injury may result in the loss of bladder sensation as well as uncontrolled and incomplete micturition. For therapeutic purposes like continence training based on biofeedback techniques and self-monitoring of patients, the knowledge of bladder filling in real time is essential [2].

Intermittent self-catheterisation is the method of choice for people with spinal cord injury and other patients suffering from neuropathic bladder to regularly empty their bladder and prevent overdistention, as the patients themselves cannot sense the urge to micturate [3]. Nevertheless, self-catheterisation is often performed even though the bladder might not yet be full or, even worse, when the urine level has already reached a critical threshold leading to renal reflux.

Therefore, “portable” bedside ultrasound devices, so-called “bladder scan” (BS) are available that enable patients to self-check their bladder volume [4–6]. However, these devices are often bulky and the user has to be trained very well to achieve a more or less reliable outcome [2, 5]. The possible integration of an automatic non-invasive measurement system into a portable device would enable self-monitoring in patients with bladder dysfunction and also enhance their mobility.

Electrical impedance tomography (EIT) is proposed as an unobtrusive cystovolumetric technique to determine bladder volume continuously [7–10]. This technique has been successfully applied in intensive care for monitoring of lung function [11–13]. For this measurement, a set of electrodes is placed around the torso and a small alternating current (AC) is injected via two of the electrodes into the body. The resulting surface voltages are measured between the remaining pairs of electrodes and provide information about the cross-sectional impedance distribution in the thoracic region and lungs, respectively.

There is no real distinction between the organ tissue of lung and urinary bladder with regard to electrical resistivity [14]. Apart from their anatomy and function, the actual difference between these organs is that the lung is normally filled with air and the bladder is filled with liquid. However, as air is a very good isolator, whereas urine has almost the same electric conductivity as the surrounding tissue, this might lead to problems in bladder volume estimation by the EIT method. When calculating volume distributions based on differences in impedances, highest accuracy is achieved when electrical impedances between the medium in the hollow organ and the surrounding tissue differ substantially. Nevertheless, first attempts showed promising outcomes and the measured impedance shows a linear correlation with bladder volume for a given urine conductivity [7].

While the EIT electrode configuration and the signal analysis algorithm are well established for estimation of the lung volume, little is known about the optimal parameters for bladder measurement. Therefore, this pilot study aims to acquire preliminary data for the evaluation of (i) the EIT method for the estimation of bladder volume in comparison to standard ultrasound methods and (ii) Four possible electrode configurations with respect to their accuracy for non-invasive cystovolumetry by EIT. The EIT data are analysed by comparison with standard ultrasound and BS measurements, which are currently the gold standard for non-invasive cystovolumetry.

## Methods

### Study design

This prospective, monocentric pilot study was approved by the board of the local Ethics committee of the Medical Faculty at the University Hospital of RWTH Aachen (Trial registration EK 169/13) and has been registered at the WHO (Clinical Registration Number: DRKS00012871).

### Participants’ characteristics

Ten healthy volunteers participated: 5 women (aged 30.6 ± 2.2 years) and 5 men (aged 33.6 ± 8.3 years). As the amount of abdominal fat may have an influence on electrical conductivity and current distribution, the body mass index (BMI) of all participants was calculated using the formula

1 $$BMI = \frac{m}{{l^{2} }}$$

with (**m**) the body mass given in kg, and (**l**) the body height in m.

Prior to the EIT study, the participants were advised to keep a drinking and micturition diary (at home) using a measurement pitcher for at least 3 × 24 h, to get baseline data on micturition volumes and frequency and to ensure normal physiology of their bladder and drinking behaviour.

### Measurement setup

**Fig. 1:**
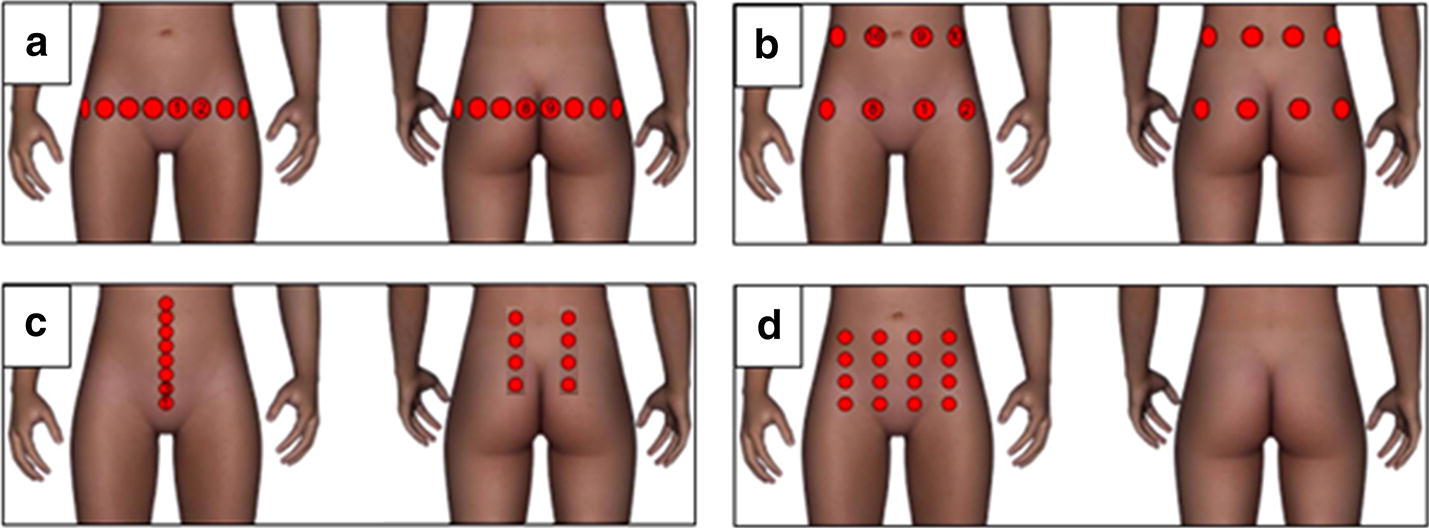
Illustration of the four different electrode configurations for EIT measurements. Each configuration contains 16 electrodes arranged as: **a** “1 × 16”, **b** “2 × 8”, **c** “Stripes” or **d** “Matrix”

For EIT measurement, 16 ECG electrodes (3 M Deutschland GmbH, Neuss, Germany) were placed on the lower abdomen and back of the participants in four different electrode configurations: (a) 1 ring of 16 equidistant electrodes = “1 × 16”), (b) 2 rings of 8 equidistant electrodes each = “2 × 8”), (c) a set of stripes = “Stripes”, consisting of a single ventral stripe of eight equidistant electrodes and two dorsal stripes of four electrodes laterally and equidistantly displaced to the right and left, respectively, and (d) a 4 × 4 ventral matrix arrangement = “Matrix”. The spot directly above the symphysis of each participant was used as lowest point for placing the first electrode. The other electrodes were applied equidistantly around the lower abdomen. Figure 1 illustrates the electrode configurations.

The “1 × 16” is the classical configuration, used in lung patient surveillance, whereas the other configurations were chosen due to their promising outcome in finite element (FEM) simulations for cystovolumetry, as has been shown by Schlebusch et al. [15].

**Fig. 2:**
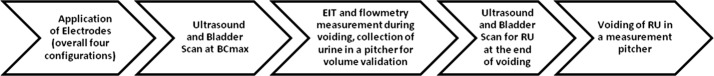
Flow chart of measurement setup

In Fig. 2 a flow chart illustrates the measurement setup for the calculation of bladder volumes as followed.

**Fig. 3:**
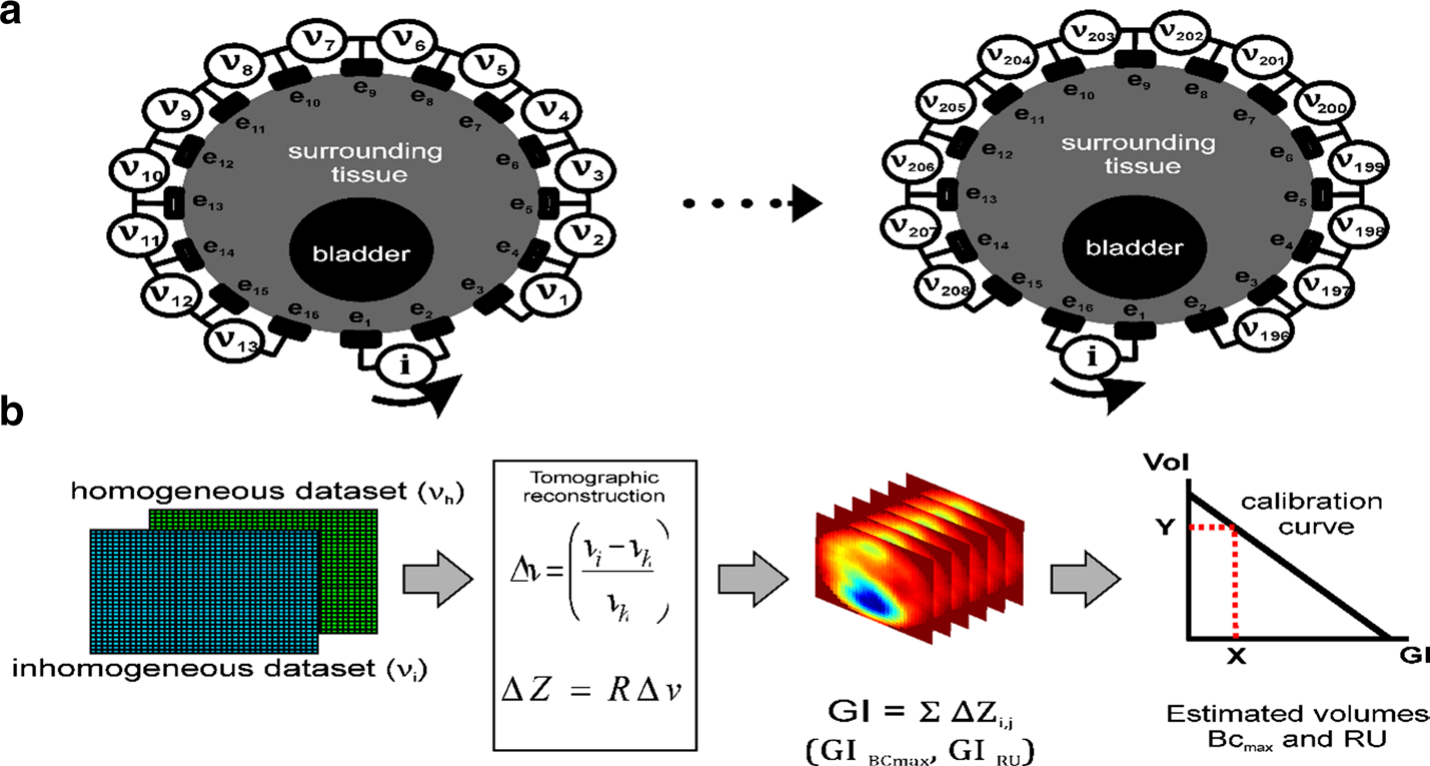
EIT measurement principle and global impedance method. **a** The 16-electrode ring arrangement is shown. Current (i) is injected between electrodes e1−e2 and the voltage measured on the remaining pair of electrodes (**a**, left). The injecting pair of electrodes is successively changed until all 16 pairs are covered (**a**, right). **b** The reference homogeneous measurement (vh: empty bladder), is subtracted from the bladder inhomogeneous measurement data (vi: BCmax), and mapped to impedance change (ΔZ) with a reconstruction matrix (*R*). A global impedance (GI) value is calculated for each EIT frame by summing every pixel value (ΔZ_i,j_). To apply the global impedance method to the estimation of BCmax and RU, at least two measurement cycles are needed. A calibration curve is generated from the first measurement cycle assuming a linear regression. The volume is estimated using the data from the second measurement cycle

Participants were advised to drink steadily. At the time point of urgency (bladder capacity maximum, BCmax), a standard ultrasound measurement using a Voluson 730 (GE Healthcare GmbH, Solingen, Germany) and a bladder scan (BS) with a portable bedside CUBEscan Biocon 500 (Medline International Germany GmbH, Kleve, Germany) ultrasound device were performed by a professional clinician for comparison with the EIT method. The bladder volume was automatically calculated by Voluson 730 based on two different methods, namely the ellipsoidal method (US Ellipsoid), and the length × width × height × correction factor 0.53 method (US L × W × H) and for bladder scan according to the procedure described in the user-manual. The ECG electrodes were then connected to the EIT Goe MF II device (Abimek, Friedland, Germany) via a 16-electrode patient cable and the participants emptied their bladder into a measuring pitcher, in a sitting position, while simultaneous recordings were made of uroflowmetry (micturition volume/time) and EIT measurements. For EIT, a current of 5 mA at 50 kHz was applied through one pair of injecting electrodes and the resulting voltage was recorded on the remaining pairs of electrodes. The pair of injecting electrodes was then successively changed and voltage measurements were repeated with all different combinations of electrodes, generating an EIT voltage frame. The standard electrode setup consists of a ring of 16 electrodes resulting in an EIT voltage frame of 13 × 16 = 208 voltage frames, from which an impedance distribution can be reconstructed. For each EIT volume estimation two measurement cycles were needed. The data recorded in the first cycle were used to generate a calibration curve for the evaluation of the EIT data of the second cycle (Fig. 3). EIT baseline measurements of maximum bladder volume (BCmax) and residual urine (RU) were taken by recording for at least 30 s before and after bladder voiding.

### Calculation of bladder volume

Volume validation was carried out by uroflowmetry consisting of a measurement pitcher, an electronic balance with a weight transducer and corresponding software (Flow, Laborie, Montreal, QC, Canada) to record the flow rate of external urinary stream as volume per unit time [ml/s] according to recommendations of the standardization committee of the International Continence Society (ICS) and urine collection in the pitcher to compare the accuracy of EIT to ultrasound and bladder scan volumetry [16]. Validation of the actual bladder volume was performed by measuring the weight of the voided urine with an electrical scale. Immediately after voiding, calculation of RU was performed via standard ultrasound and bladder scan. Afterwards, the voided RU was collected in the weight measurement pitcher, without EIT measurement. The BCmax at the point of urgency was determined as micturition volume plus RU from the measurement pitcher. This procedure was performed twice for each participant and for each electrode configuration.

Results from the measurement pitcher (in grams) were converted into a volume using a conversion factor known from the literature, i.e. 1.02 g/ml [17]. Because multiplication of the urine weight by this factor showed very good correlation with the urine volume (99.9%), for further measurements only the pitcher volume was used for validation of the measurements. For EIT volume estimation, one of the voiding measurements of each volunteer was used as calibration data for the estimation of BCmax and RU from the second measurement.

### The EIT method

Electrical impedance tomography reconstruction is an ill-posed, nonlinear inverse problem [18]. A reconstruction matrix is calculated (**R**) for each electrode configuration using the widely accepted Graz consensus reconstruction algorithm for EIT (GREIT) [19]. Moreover, EIT is reconstructed as a differential measurement,

2 $$\Delta v = \left( {\frac{{v_{i} - v_{h} }}{{v_{h} }}} \right)$$

3 $$\Delta {\mathbf{Z}} = {\mathbf{R}}\Delta v$$

where a reference EIT measurement of the homogeneous medium (**ν**_h_), is subtracted from the measured surface voltage vector of the inhomogeneous medium (**ν**_i_). In this case, the homogeneous medium (**ν**_h_) is the empty bladder and (**ν**_i_) is the recorded signal at BCmax. **R** is a 1024 × 208 linear reconstruction matrix that maps the normalised voltage difference measurements (Δ**ν**) to an impedance image matrix (Δ**Z**). Therefore, the reconstructed image consists of 1024 = 32 × 32 pixels. It is assumed that the reactance of urine is negligible and the resistance changes in the measured signal are only caused by the local resistance changes due to the change in bladder volume. Data analysis was done using MATLAB (v.2015b) and the reconstruction matrix calculated using the EIDORS framework (v.3.8) [20].

### EIT cystovolumetry

**Fig. 4:**
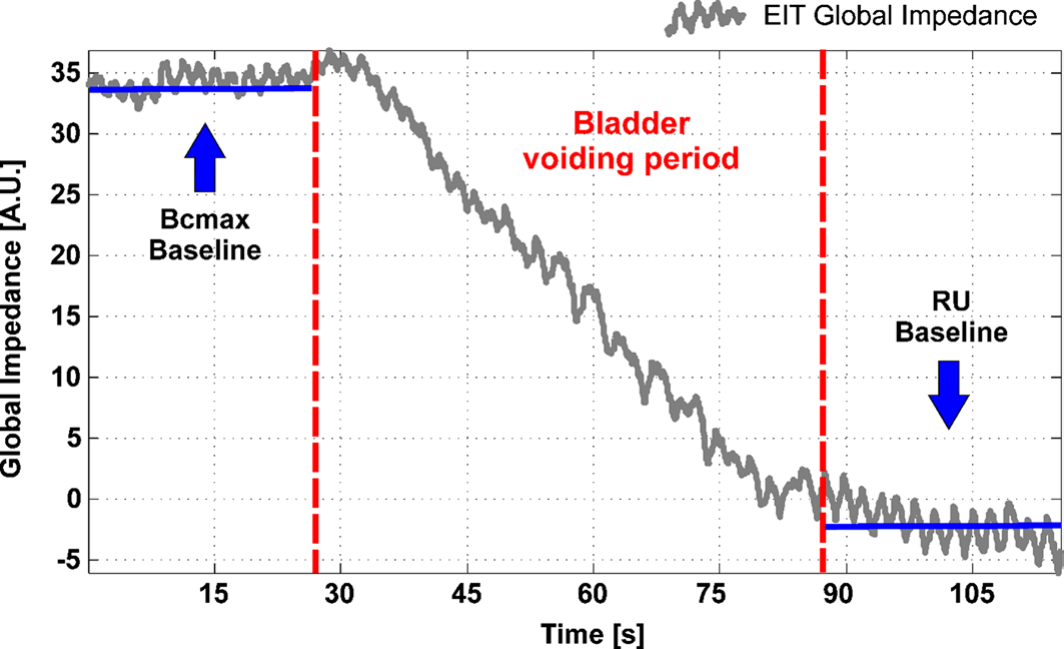
Example of a global impedance curve from a voiding trial. The measurement is divided in three segments: a BCmax baseline recording (left), a bladder voiding period (centre), and a residual urine (RU) baseline recording (right). BCmax and RU are averaged in the corresponding segments and correlated to the pitcher volumes to generate a Volume vs. Global Impedance linear regression, as depicted in Fig. 3

**Fig. 5:**
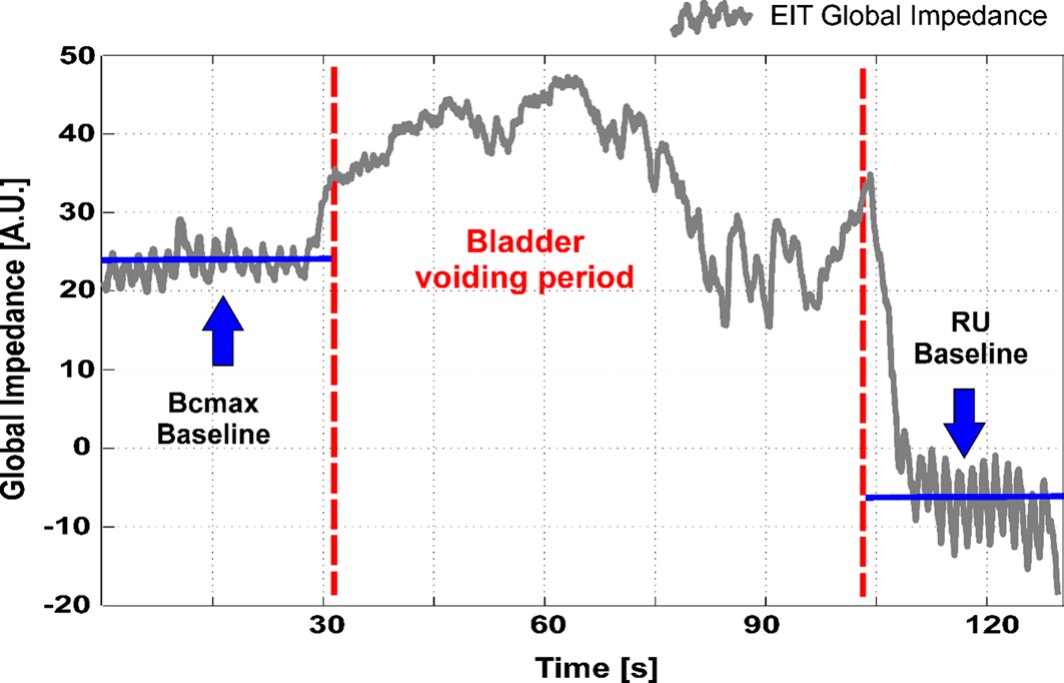
Example of a global impedance curve from a voiding trial with artefacts. The curve is not linear during the bladder voiding period (compare with Fig. 4). This is probably caused by artefacts due to involuntary body movements during micturition in a sitting position. Abdominal muscle contraction and relaxation may cause the drastic impedance changes observed both at the beginning and at the end of the voiding period. However, baseline recordings of BCmax and RU are still extractable

EIT may provide a method to measure bladder volume continuously and non-invasively. A strong linear, negative correlation has been shown between the measured lower abdomen global impedance and bladder volume [7]. With increasing volume of the bladder, the measured lower abdomen impedance decreases linearly. This relationship is reasonable, due to the fact that urine conductivity is usually higher than the conductivity of the surrounding tissue. For the estimation of bladder volume, the global impedance method was used. A tomographic reconstruction matrix was calculated for each of the four electrode arrangements (Fig. 1). By multiplying the corresponding reconstruction matrix with the measured voltage data an image of the impedance distribution is obtained, at a sampling rate of 13 EIT frames per second. Each pixel corresponds to a relative impedance value. The global impedance per EIT frame results from adding all the pixel values in an image. After an initial calibration measurement of two known volumes, i.e. empty and maximally full bladder, a relationship between impedance and bladder volume is obtained for each trial participant, assuming a linear correlation. With this assumption, a bladder volume can be estimated for later measurements by linear regression fitting (Fig. 3b). Therefore, and in order to acquire more reliable full and empty bladder measurement data, an extended recording was conducted both before (BCmax) and after voiding (RU), and the impedance values were averaged over this time. Figure 4 shows an example of an EIT global impedance curve for a bladder voiding trial. In the present study, the EIT measurement during micturition was strongly affected by abdominal muscle contractions and body movements such as leaning forwards or backwards in the sitting position, as depicted in Fig. 5. Therefore, the EIT data during the voiding period were not included in the linear regression calculation. Since at least two measurements cycles per participant were performed, the data from the first voiding measurement cycle were used for calibration and the data from the second voiding measurement cycle for estimation of the bladder volume. However, since not all participants strictly adhered to the protocol and were unable to void RU as they had no RU by ultrasound or could not micturate, some of the data had to be excluded from the analysis. Finally, 12 measurements were available for the 1 × 16 electrode arrangement, and 16 measurements were available for the 2 × 8, Stripes and Matrix arrangements, each consisting of a calibration and a measurement cycle. It is important to mention that a certain error in the assumed actual RU volume might be present due to the own urine production of the participants between the RU–US measurements and the final voiding. Although the time interval between both procedures was kept short, the participants’ increased diuresis due to the high liquid intake may cause a deviation. A second US measurement after RU voiding was not performed.

### Statistical analysis

For statistical analysis the Shapiro–Wilk test was performed to test for normal distribution. Because the US, BS and EIT data showed a normal distribution, Student’s *t* test was used to determine significant differences between the measurement methods and the EIT configurations. A p-value < 0.05 was considered to indicate statistical significance. The software used was OriginPro (2017G, Origin Lab Corporation, Northampton, USA). If not indicated elsewhere values are shown as mean values plus/minus standard deviation.

## Results

### Participants’ baseline data

Female participants had normal BMI values (mean 22.1 ± 3.0 kg/m^2^) whereas the values of the men tended towards overweight (mean 25.2 ± 3.9 kg/m^2^), but the difference for both gender was not significant. Mean baseline micturition values surveyed via the bladder diary were within the range as described for healthy men and women [21]. Here, the mean micturition frequency in women was 8.3 ± 1.2/day and in men was 6.4 ± 2.2/day (7.1 ± 2.1/day for men and women combined). Mean micturition volume was 284 ± 93 ml in women and 317 ± 45 ml in men (combined: 305 ± 63 ml). This results in a total volume of 2337 ± 627 ml/day for women and of 1975 ± 778 ml/day for men (combined: 2110 ± 702 ml).

### Measurement cycles

#### Comparison of baseline and study data

**Fig. 6:**
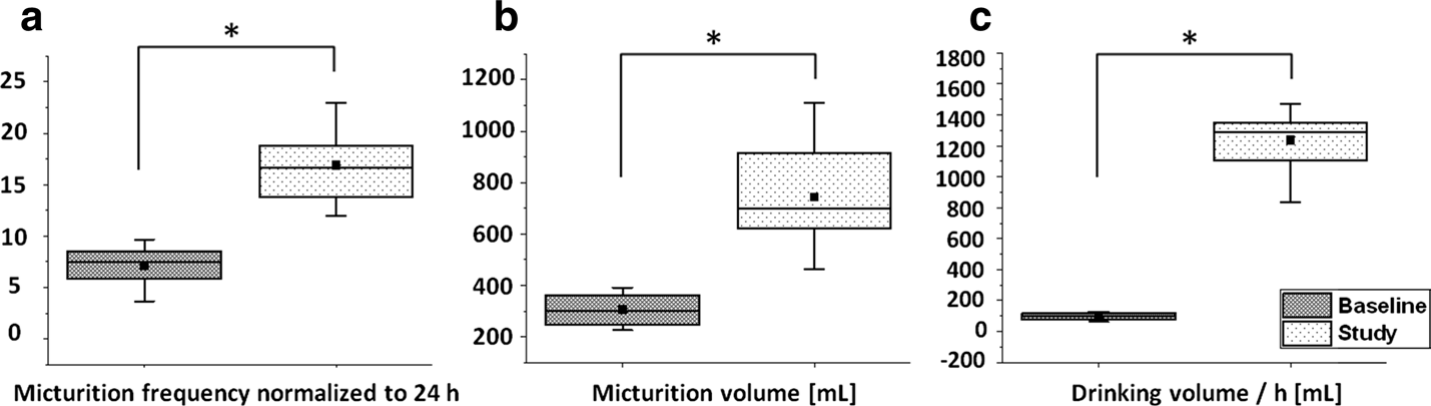
Micturition frequency/volume and drinking volume of the participants (sexes combined). Comparison of the Baseline and Study data revealed that participants had significantly increased: **a** micturition frequency and **b** volume, which resulted from **c** an excessive drinking behaviour during the study days. P < 0.05 indicates a significant difference and is marked with an asterisk

During the study visits, all participants were asked to considerably increase their drinking volume to acquire at least two measurement cycles per study visit, which resulted in increased micturition frequency and volume as depicted in Fig. 6. Women and men had 0.7 ± 0.2 micturitions per hour (without RU) which amounts to 17.0 ± 4.4/day. Again, the actual BCmax was validated by collection of the micturated urine during the EIT-measurement and the micturated RU after the EIT-measurement in a measurement pitcher. Mean values of BCmax in women were 804 ± 228 and 683 ± 170 ml in men (combined: 743 ± 200 ml). None of the participants showed any signs of RU at home. During the study, RU, collected after the final US and BS measurement in the measurement pitcher, was 171 ± 76 ml in women and in men 134 ± 51 ml (combined: 152.1 ± 64 ml). However, in addition to changes in micturition volume and frequency, also the drinking behaviour changed. At home, women drank 2374 ± 685 ml per 24 h and men drank 2299 ± 572 ml per 24 h (combined: 2327 ± 568 ml per 24 h) which equals a fluid intake of 99 and 96 ml/h, respectively (combined: 97 ml/h). During the measurement days, liquid ingestion was 1254 ± 180 ml/h for women and 1218 ± 228 ml for men (combined: 1236 ± 195 ml). Because the differences between men and women were not significant, further evaluation of the comparison between US and EIT was performed with men and women combined. For clarification, these data are presented in Fig. 6.

### Evaluation of measurement accuracy of US, BS and EIT

**Fig. 7:**
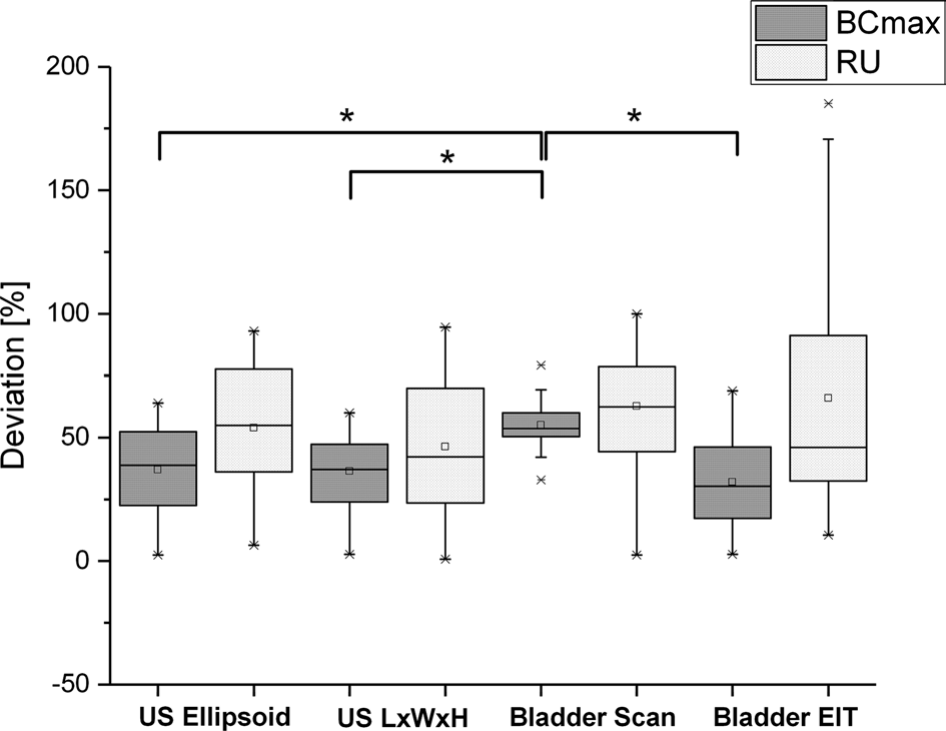
Deviation of the different measurement tools for cystovolumetry compared to the actual bladder volume, validated by urine collection in the measuring pitcher. For BCmax, the two US methods and the EIT method are similar but, nevertheless, show a significant offset from the actual bladder volume. However, BS has a significantly higher error compared with the other three methods. For estimation of residual urine (RU), all four methods (including EIT) showed decreased accuracy. P < 0.05 indicates a significant difference and is marked with an asterisk

The volume calculated by the ultrasound devices was always lower than the actual BCmax or RU. The results are presented as the mean deviation with respect to actual urine volume collected in the measurement pitcher for US-Ellipsoid, US-L × W × H, BS and EIT in the estimation of BCmax and RU. The mean error was normalised with respect to BCmax (743 ± 200 ml) and RU (152.1 ± 64 ml) as measured with the measurement pitcher. For the estimation of BCmax, the US-Ellipsoid (37 ± 17%) and the US-L × W × H (36 ± 15%) demonstrated a similar performance, whereas BS (55 ± 9%) had a significantly higher average error. In contrast, EIT (32 ± 18%) showed a better accuracy than the standard sonographic measurement techniques. However, for the estimation of RU, both EIT (72 ± 58%) and BS (63 ± 24%) showed comparable but also the highest errors, whereas the error for US-Ellipsoid and US-L × W × H was lower, i.e. 54 ± 25% and 46 ± 24%, respectively (Fig. 7).

### Evaluation of electrode configurations

**Fig. 8:**
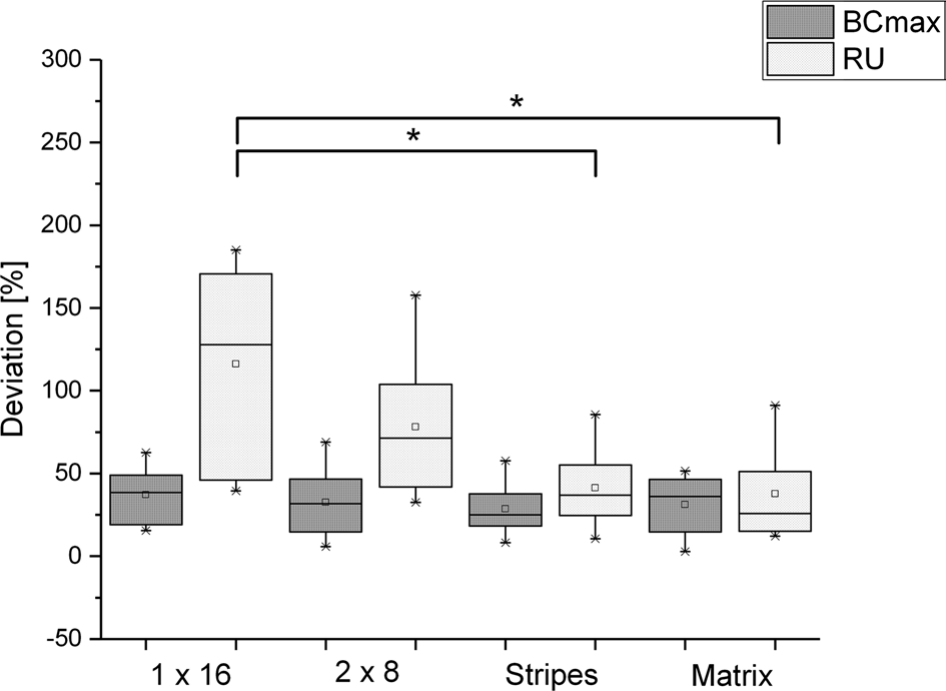
Comparison of the electrode configurations (given as deviation in percent) to the actual bladder volume. All four configurations show comparable accuracy for estimation of BCmax. For estimation of residual urine (RU), 1 × 16 is significantly inferior to the Stripes and Matrix configuration. 2 × 8 shows a similar inferior trend, although not significant

Comparison of the four electrode configurations revealed that all configurations were comparable to the US methods for measurement of BCmax. However, this was not true for RU. Here, the Matrix (38 ± 27%) and Stripes (41 ± 25%) configurations showed significantly better accuracy than 1 × 16 (116 ± 62%). The 2 × 8 (78 ± 43%) configuration shows a similar inferior trend compared to the Matrix and Stripes, but the difference was not significant (Fig. 8). This also explains the large error of EIT–RU in Fig. 7, as the measurement data of all four electrode configurations were combined in order to compare Bladder EIT with US and BS.

## Discussion

As shown by Leonhardt et al. EIT is a promising tool for the estimation of bladder volume in individuals with spinal cord injury [7]. However, in individual patients, the differing amounts of body fat can alter the results or impede cystovolumetric measurements [6]. Therefore, for this study, data on BMI were acquired to align BMI data with the impedance data, if needed. However, since the healthy participants in this study showed no significant differences in BMI there was no need to create a correlation factor for this parameter. Nevertheless, this might prove to be an issue in future studies.

During the study days, the non-physiological liquid ingestion of the (highly motivated) participants lead to a raised renal function resulting in increased diuresis [21]. Comparison of the micturition diaries with micturition volume revealed that, in normal life, none of the participants had an abnormal drinking or micturition behaviour. Nevertheless, the resulting high bladder volumes and small amounts of RU allowed to test the standard US and BS measurements, together with the new EIT method, under extreme conditions.

Although some studies have reported the superiority and reliability of ultrasound as a clinical tool, others have reported problems related to the measurement of bladder volume [22–25]. According to Carter et al., the ellipsoid US remains the best method for use in the urological clinic. However, in the present study this was not apparent since the L × W × H showed high similarity to the ellipsoid US [26]. With respect to the validation of EIT cystovolumetry in our study, the real voided urine volume had to be used to correlate these data to our impedance signals, because the ultrasound data were not reliable.

Nevertheless, this study allowed us to compare standard US techniques with the proposed EIT-based cystovolumetry. The US methods show a considerable error for estimation of both BCmax and RU, whereas bladder EIT shows slightly better accuracy than US and BS for BCmax estimation. For the estimation of RU, all methods show decreased accuracy compared to the estimation of BCmax, and EIT, when combining all four configurations tested, shows a higher error than the standard US and BS methods. A possible explanation for the reduced sensitivity of the 1 × 16 and 2 × 8 EIT configurations to low bladder volumes is that the EIT electrode positions are fixed on the body and, at lower volumes, the bladder falls below the electrode plane behind the symphysis thereby lowering the sensitivity of the EIT system to bladder volume changes. Therefore, the electrode configuration to be used in future should cover a larger transversal volume and enable measurement of all levels of bladder filling, as realized by the “Matrix” or “Stripes” configurations.

Although our aim was a continuous measurement of the bladder volume during micturition with the EIT method, we encountered major measurement problems in form of movement artefacts and additional stomach pressure due to abdominal muscle contraction which caused volume-independent impedance changes (see Fig. 5). Therefore, we could only compare the full with the voided bladder. The impact of these comparably small muscle tensions must be taken into account in the case that EIT electrodes are integrated into fabric [27, 28].

These are additional outcomes that, on the one hand, show the limitations of bladder volume estimation itself and the need for improvement of the standard devices or development of new devices. On the other hand, they can help in the development of our medical EIT device and the specifications for a new cystovolumetry algorithm.

## Conclusions

The use of standard ultrasound as a reference method for the development of a new EIT bladder volume device has to be neglected, as there was a large offset between the measurements and actual bladder volume. Volume validation by means of a measurement pitcher and a weight scale is recommended as reference.

EIT, in comparison to standard ultrasound-based measurement systems, shows considerable potential as a cystovolumetry system. However, before being used in everyday applications and as a continuous method, its accuracy needs to be improved by minimizing the negative influence of movement artefacts and by optimising the electrode configuration and calibration.

## Abbreviations

BCmax: maximum bladder capacity
BMI: body mass index
BS: bladder scan
EIT: electrical impedance tomography
L × W × H: formula calculated from length, width and height
US: ultrasound
RU: residual urine
